# Two novel heterozygote mutations of ATM in a Chinese family with dystonia-dominant ataxia telangiectasia and literature review

**DOI:** 10.3389/fped.2023.975696

**Published:** 2023-03-15

**Authors:** Zhi-Jun Liu, Ya-Ling Wang, Yan Xu

**Affiliations:** Department of Neurology, Union Hospital, Tongji Medical College, Huazhong University of Science and Technology, Wuhan, China

**Keywords:** ataxia-telangiectasia, targeted exome-sequencing, ATM, dystonia, Chinese

## Abstract

**Background:**

Ataxia-telangiectasia (A-T) is an autosomal recessive disorder with high clinical heterogeneity. A-T may present in complicated variable forms, including classic A-T and milder form of AT. Contrary to the classic A-T, the milder form does not present the cardinal features of A-T such as ataxia and telangiectasia. A few *ATM* mutations have been reported in variant A-T cases manifesting isolated generalized or segmental dystonia without any signs of classical A-T.

**Methods:**

An A-T pedigree with predominant dystonia was collected. Genetic testing was performed by targeted panel of genes involved in movement disorders. The candidate variants were further confirmed by Sanger sequencing. We then reviewed previously published literatures of genetically confirmed A-T cases with predominant dystonia and summarized the clinical characteristics of dystonia-dominant A-T.

**Results:**

Two novel *ATM* mutations, p.I2683T and p.S2860P, were identified in the family. The proband presented isolated segmental dystonia without any signs of ataxia and telangiectasias. We reviewed the literatures and found that the patients with dystonia-dominant A-T tend to have a later-onset and slower progression of the disease.

**Conclusion:**

To our knowledge, this is the first report of A-T patient with predominant dystonia in China. Dystonia may appear as one of the predominant manifestations or initial symptom of A-T. Early ATM genetic testing should be considered for those patients with predominant dystonia, despite without accompanying ataxia or telangiectasia.

## 1. Introduction

Ataxia-telangiectasia (A-T) is a rare autosomal recessive disorder characterized by progressive cerebellar degeneration, telangiectasia, immunodeficiency, radiosensitivity, recurrent respiratory tract infections, and increased risk of cancer (1). Most people with A-T develop the disease in early childhood and die from malignancies or respiratory failure in the second or third decade of life (2).

**Figure 1:**
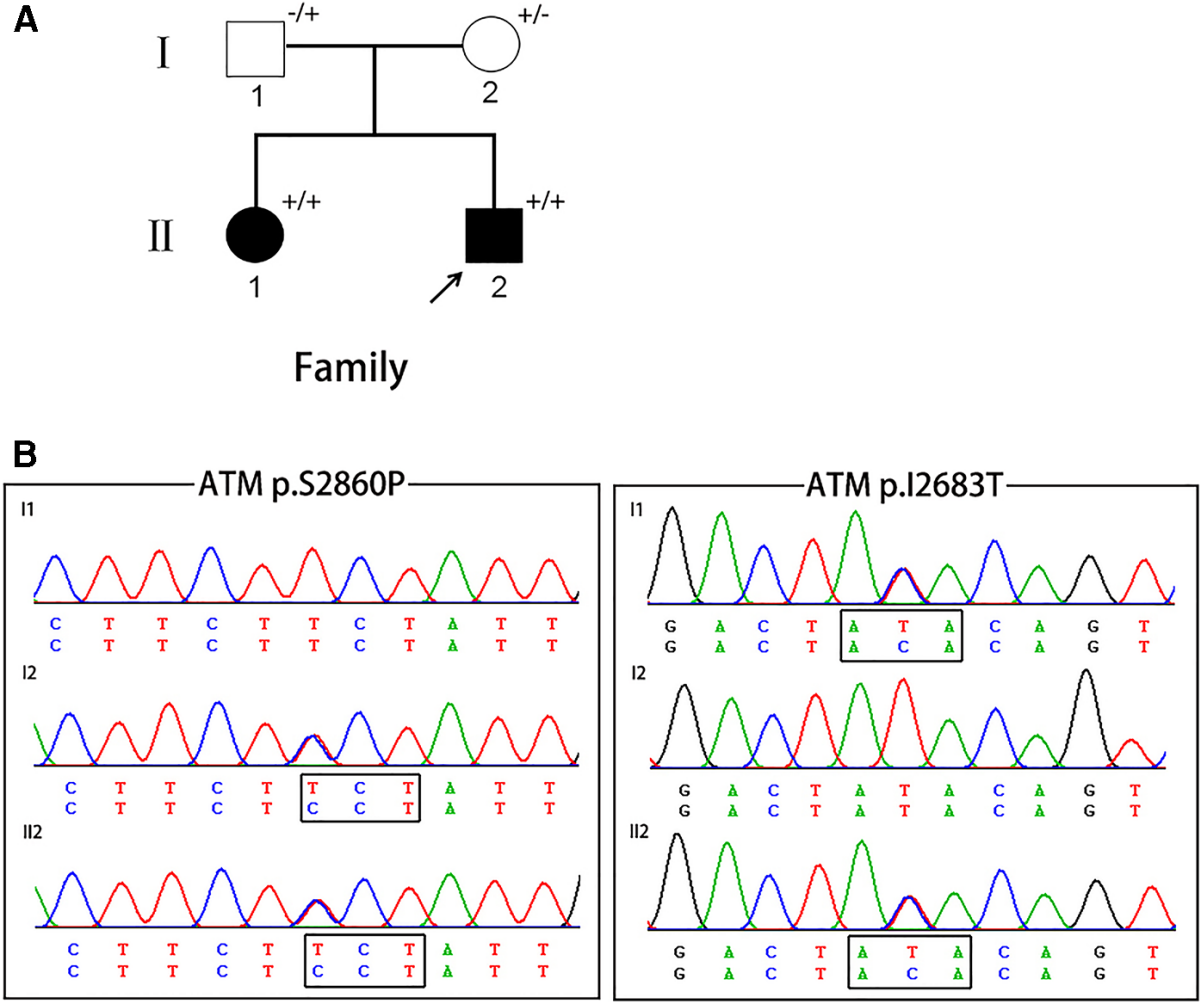
(**A**) The pedigree of A-T patients in present study. Squares indicate males; circles indicate females; filled symbols indicate affected individuals; arrows indicate the probands; “+/+” indicate two mutant alleles; “+/−” or “−/+” indicate mutation occurring in one of two alleles. (**B**) Chromatograms of the proband and the proband's parents.

A-T may present in complicated variable forms, including the classic A-T which is caused by truncating ataxia telangiectasia mutated (ATM) mutations in both alleles leading to total loss of ATM kinase activity and milder form of AT (also known as variant A-T) often associated with missense or leaky splice mutations (3). Patients with classic A-T usually develop an ataxic gait in early childhood and become wheelchair dependency by adolescence (4). Contrary to the classic A-T, the milder form does not present the cardinal features of A-T, such as ataxia, telangiectasia, and immunodeficiency. Other characteristics of movement disorders including chorea, myoclonic jerks, resting tremor, and dystonia are well recognized as the presenting manifestations of variant A-T (5). A systematic review of movement disorder phenomenology and systemic features in A-T patients have found that 89%(186/210) cases manifested dystonia and 18%(24/130) presented dystonia as their initial manifestations of A-T (6). Notably, a few *ATM* mutations have been reported in variant A-T cases manifesting as isolated generalized or segmental dystonia without any signs of classical A-T (7).

A-T is caused by mutations in the *ATM* gene which plays a critical role in pathway regulating DNA double-strand breaks (DSBs) repair. To date, more than 400 disease-related *ATM* mutations have been identified in patients with A-T. Among these ATM mutations, the majority (about 70%) are truncating changes resulting in premature translation termination (2).

In this study, we reported a Chinese A-T family with two novel compound heterozygote mutations in the *ATM* gene. To our knowledge, this is the first report of A-T patient with predominant dystonia in China. Our findings broaden the molecular and clinical spectrum of Chinese A-T patients.

## 2. Methods

### 2.1. Participants

The pedigree was collected from the Neurology Department of Wuhan Union hospital. DNA samples were obtained from all the participants, including the proband, his affected older sister and their parents. Magnetic resonance imaging (MRI) of brain and cervical, ultrasound of heart and abdominal, electroencephalogram (EEG), and blood biochemical analysis (detection of *α*-fetoprotein (AFP), immunoglobulin(Ig), and ceruloplasmin) were conducted. An additional 200 normal individuals without any neurological disorders were included as controls. Written informed consents were obtained from all participants. This study was approved by the ethics committee of Wuhan Union Hospital.

### 2.2. Targeted exome-sequencing

Genomic DNA was extracted from peripheral blood samples by DNA extraction Kit (Qiagen, Germany). A panel was designed to cover 101 genes associated with movement disorders (Supplementary Table S1). Deep sequencing was performed using Illumina Hiseq2000 system (GrandOmics Biosciences Co, China). Sequencing analysis was performed as we described previously (8). In order to screen for large deletions or duplications of Dopa-responsive dystonia (DRD) associated genes (*GCH1, TH,* and *SGCE*), the Multiplex ligation-dependent probe amplification assay (MLPA) analysis was performed by reference to the method previously reported (9).

### 2.3. Sanger sequencing

Sanger sequencing was used to validate the candidate variants after data analysis. Forward and reverse primers were designed to amplify the fragments covering the variant sites. The fragments were sequenced on ABI 3530xL DNA Sequencer. Co-segregation analysis was conducted through screening for the confirmed variants in the proband's older sister and parents.

## 3. Results

### 3.1. Genetic findings

No large deletions or duplications of *GCH1, TH,* and *SGCE*, were found in the proband. About 94.5% of the target bases were covered with at least 50X, and the mean depth of coverage for all target regions was 170. After filtering, two novel missense variants in *ATM*, c.T8048C (*p*.I2683T) and c.T8578C (*p*.S2860P), were identified in the proband (Figure 1). The two novel variants were compound heterozygous in the proband and his affected older sister, and were heterozygous in their unaffected parents. These two variants were absent from major public variant databases (1000G frequency, ESP6500, and ExAC frequency) and our 200 normal individuals. The two variants were predicted as harmful effects by the SIFT, PolyPhen-2, and MutationTaster prediction software. According to the ACMG standards and guidelines, these two variants were classified as likely pathogenic variants.

### 3.2. Clinical manifestations

The proband (**II:2**), a 15-year-old boy, was delivered by forceps with no birth complications and had a normal childhood development milestones. He started walking around one year of age and had normal speech. He presented at our clinic complaining of neck pain and stiffness since 9 years old, which progressively worsened over the period of 2–3 years. He developed mild head shake at age 12. Then he experienced episodes of involuntary turning of the head to the left and cervical hyperextension, postural hand tremor, and blepharospasm at age 13. On examination at age 15, he had severe dystonia with dominant craniocervical involvement. The jerky right torticollis, postural hand tremor, and choreiform facial movements were present. Eye movements were normal with normal saccades responses and ocular pursuit movements. He had normal muscle power and reflexes, and a slightly increased muscle tone in left upper limb. Sensory examination was unremarkable. There were no cerebellar signs. His cognitive function remains intact, with a Mini-Mental State Exam (MMSE) and Montreal Cognitive Assessment (MoCA) score of 28/30 and 25/30, respectively. Eye ophthalmic examination was unremarkable.

His older sister (**II:1**) was a 19-year-old girl and experienced similar symptoms since age 12 years. She noted her first symptom of mild involuntary head shake while taking a photo at the age of 12 years. As the disease progressed, she gradually developed jerky right-sided torticollis, postural hand tremor, oromandibular dystonia. She had recurrent episodes of transient global amnesia, which occurred 2–3 times per year. Her examination was further marked by severe cervical and oromandibular dystonia, slightly increased muscle tone in left upper limb, and postural hand tremor. The deep tendon reflexes were all absent, with flexor plantar responses. She showed normal saccades and ocular pursuit movements. Her cognitive function was unremarkable.

There was no known consanguinity in the family. The proband's grandmother had a gastric cancer and died at age 50. The detailed clinical results are summarized in Table 1. The serum AFP levels were significantly increased in the two patients, whereas serum levels of IgG and IgA was significantly decreased. Other laboratory tests including cholesterol, creatinine alkaline, lactate dehydrogenase (LDH), and ceruloplasmin were all normal. The brain and spinal cord MRI, ultrasound of heart and abdominal, and electroencephalogram (EEG) were unremarkable.

**Table 1:** Summary of clinical manifestations and findings of variant AT cases with predominant dystonia.

Families	Present study	Simonin et al. (10)	Carrillo et al. (11)	Saunders-Pullman et al. (12)			Meissner et al. (13)	Cummins et al. (14)	Kuhm C et al. (15)	Lohmann et al. (16)	Necpál et al. (7)	Ganguly et al. (17)	Lnu et al. (18)
Origin	Central-southern China	France	Indian	Canadian Mennonite	Canadian Mennonite	Canadian Mennonite	France	United Kingdom	Germany	Germany	Indian	Canadian Mennonite	Indian
Affected	2 (1M, 1F)	4 (2M, 2F)	1(F)	6 (4M, 2F)	2 (1M, 1F)	4 (1M, 3F)	3 (2M, 1F)	1(M)	1(M)	3(M)	1(F)	1(F)	1(M)
AAO, y	9,12	12–20	15	1–16	11–20	1–12	Adolescence to 18	2	Childhood	1–5	12	Early childhood	6
Dystonic features	Craniocervical	Legs, 3/4	Craniocervical	Neck, arms, legs	Neck	Neck, legs	Generalized	Upper and lower limbs	Generalized	Generalized	Generalized	Neck, upper limb	Neck
Chorea	1/2	2/4	No	5/6	1/2	4/4	0/3	No	No	2/3	No	No	No
Cerebellar ataxia	No	4/4	No	No	No	No	1/3	No	No	0/3	No	No	No
Telangiectasia	No	No	Yes	No	No	No	0/4	No	No	0/3	No	No	Yes
Malignancy	No	2/4	No	4/6	0/2	3/4	0/3	No	No	0/3	No	No	NA
Intelligence decline	No	1/4	No	NA	NA	NA	NA	No	NA	NA	NA	NA	NA
Decreased ATM protein	NA	NA	Yes	NA	NA	2/4	2/3	Yes	NA	NA	NA	NA	NA
Elevated AFP level	2/2	2/2	Yes	NA	NA	2/4	NA	Yes	Yes	1/3	Yes	Yes	Yes
Decreased IgA, IgG	2/2	1/2	NA	NA	NA	NA	NA	NA	Yes	NA	No	No	Yes
Additional features	Transient global amnesia	Axonal neuropathy	No	No	No	Sensorimotor neuropathy	Atrophy of lower third of both thighs	No	Delayed motor development, speech and swallowing difficulties	Slurred speech, migraine	Pneumonia, bronchitis, pharyngitis, oligoarthritis	Delayed motor development	No
Brain MRI	Normal	NA	Mild cerebellar atrophy	NA	NA	Normal	NA	Normal	Normal	Normal	NA	Normal	NA
Family history	Yes	Yes	No	Yes	Yes	Yes	Yes	No	No	Yes	No	No	No
ATM Mutations	c.T8048C (*p*.I2683T) and c.T8578C (*p*.S2860P)	c.7271T > G (*p*.V2424G) and c.193delC(*p*.Q65Rfs*11)	c.590G > A (*p*.197G > E)	c.620° C > A (*p*. A2067D)	c.6200C > A(*p*.A2067D)	c.6200C > A(*p*.A2067D)	c.6679C > T (*p*.R2227C) and c.572T > A (*p*.I191N)	c.8266A > T(K2756*) and c.743G > T (*p*.R248l)	c.8147T > C (*p*.V2716A) and c.8578_8580delTCT(*p*.S2860del)	*p*.G301Vfs*19 and c.8147T > C(*p*.V2716A)	c.5573G > A (*p*.W1858*) and c.6154G > A (*p*.E2052K)	c.6200C>A(p.A2067D) and c.5932G>T(p.E1978*)	c.67C > T(p.R23*)

AAO, age at onset; y, year; AFP, *α*-fetoprotein; IgA, immunoglobulin A; IgG, immunoglobulin G; NA, not assessed.

## 4. Discussion

In this study, we present a Chinese A-T family with two compound heterozygous mutations of ATM presenting with atypically craniocervical dystonia and also present a review of the literatures on the variant A-T cases with dystonia predominance. No signs of ocular motor apraxia, cerebellar ataxia, as well as telangiectasia were observed in the case.

Dystonia, occurring either in isolation or in combination with other clinical features, has been described in variant A-T patients. Dystonia affecting the neck, head, trunk and limbs, may be present as initial symptoms in about 18%(24/130) of A-T patients (6). The present case showed obviously isolated segmental dystonia without any signs of ataxia and telangiectasias. After reviewed previously published literatures of genetically confirmed A-T cases with predominant dystonia, we found that dystonia-dominant A-T cases may have a significantly different clinical picture compared to the typical A-T. In total, 13 pedigrees including 30 patients with *ATM* mutations were reviewed (7, 10–18) (Table 1). The median age of onset was 10.67 years, ranging from 1 to 20 years old. The group of dystonia-dominant A-T cases showed isolated dystonic features or accompanied by choreoathetosis, tremor and/or myoclonic jerks, but without involvement of ataxia and telangiectasias. Other phenotypes including peripheral neuropathy, delayed motor development, speech and swallowing difficulties, could also be seen in the group. A few patients also experienced recurrent pneumonia and increased risk of malignancy. Overall, the patients with dystonia-dominant A-T tend to have a later-onset and slower progression of the disease. However, the relationship between dystonic phenotype and particular ATM genotype in variant A-T is not well understood. As previously reported, mutations in exon 42 of the *ATM* gene may predispose to dystonia-dominant A-T, which is needed to be confirmed in further analysis (19). Notably, the transient global amnesia observed in the proband's old sister is a relatively rare symptom and the first description in patients with A-T. Further investigation and analysis of amnesia presentation in patients with A-T is required.

In line with previous study (1), significantly elevated AFP levels and decreased serum levels of immunoglobulin (IgG and IgA) were observed in this family. Moreover, previous studies revealed that elevated level of IgM was indicative of a distinct and more severe phenotype (20). IgM level was normal in the two patients, which suggests the mild phenotype observed in the present cases. Unfortunately, we were not able to obtain tissue to perform analysis of ATM protein expression with this family.

The neuropathological hallmark of A-T is cerebellar Purkinje cell loss, followed by cerebellar symptoms and degeneration (21). The exact pathogenesis of dystonia in A-T remains unclear. A review of pathological findings in 17 patients with A-T and movement disorders found that neuronal loss occurs in the basal ganglia and brain stem nuclei (22). Previous positron emission tomography (PET) study revealed reduced glucose metabolism in the cerebellum of A-T patients and increased metabolism in the globus pallidus which often results in decreased motor performance (23). Additional studies are required to assess the association of dystonia with cerebellum and basal ganglia degeneration in A-T patients.

In conclusion, our findings expand the clinical and molecular spectrum of dystonia-dominant A-T in China. Dystonia may appear as one of the predominant manifestations or initial symptom of A-T. Isolated dystonia without any signs of ataxia or telangiectasias may result in delay in the diagnosis of A-T. Early *ATM* genetic testing should be considered for those patients with predominant dystonia, despite without accompanying ataxia or telangiectasia.

## Data Availability

The datasets presented in this study can be found in online repositories. The names of the repository/repositories and accession number(s) can be found in the article/**Supplementary Material**.
